# Dynamics of Bone Disease Biomarkers Dickkopf-1 and Sclerostin in Patients with Multiple Myeloma

**DOI:** 10.3390/jcm12134440

**Published:** 2023-07-01

**Authors:** Vladimir Gerov, Daniela Gerova, Ilina Micheva, Miglena Nikolova, Galya Mihaylova, Bistra Galunska

**Affiliations:** 1 Clinic of Hematology, “St. Marina” University Hospital, 9010 Varna, Bulgaria; ilinamicheva@gmail.com; 2 Department of Clinical Laboratory, Faculty of Medicine, Medical University-Varna, 9002 Varna, Bulgaria; dgerova@gmail.com; 3 Second Department of Internal Diseases, Faculty of Medicine, Medical University-Varna, 9002 Varna, Bulgaria; 4 Department of Biochemistry, Molecular Medicine and Nutrigenomics, Faculty of Pharmacy, Medical University-Varna, 9000 Varna, Bulgaria; miglena.todorova@mu-varna.bg (M.N.); galya.mihaylova@mu-varna.bg (G.M.); galunska@mu-varna.bg (B.G.)

**Keywords:** multiple myeloma bone disease, Dickkopf-1, sclerostin

## Abstract

Dickkopf-1 (DKK-1) and sclerostin are essential Wnt/β-catenin pathway inhibitors, playing an important role in multiple myeloma bone disease (MBD). We aimed to examine the serum DKK-1 and sclerostin variations in newly diagnosed multiple myeloma (NDMM) patients at diagnosis and in the course of therapy, including autologous stem cell transplantation (ASCT). This study included 41 NDMM-patients and 33 controls. MBD was assessed by whole-body low-dose computed tomography. DKK-1 and sclerostin were assayed by commercial ELISA kits. At diagnosis, NDMM-patients revealed significantly higher DKK-1 and sclerostin values (*p* < 0.0001), showing dependence on disease stage (lowest in ISS-I and highest in ISS-III: *p* < 0.0012 and *p* < 0.025, respectively, for both proteins). Bone lesions revealed significant positive correlation with both DKK-1 (*p* < 0.05) and sclerostin (*p* < 0.0001). In the course of therapy, significant reduction, more prominent after ASCT, was observed for both parameters in each treatment point compared to the baseline (*p* < 0.0001). Markedly lower sclerostin (*p* < 0.01) and DKK-1 (*p* < 0.05) values were observed in patients with complete and very good partial response compared to those with partial response, stable, or progressive disease. Sclerostin and DKK-1 in NDMM patients reflect the MBD severity and the effect of therapy. Both proteins could represent a novel tool for better disease monitoring and effectiveness of therapy.

## 1. Introduction

Multiple myeloma (MM), characterized by clonal proliferation of plasma cells, is one of the most frequent hematological malignancies [1], of which incidence has been increasing uniformly since 1990 [2]. The major feature of symptomatic MM is bone disease, affecting significantly the patient’s quality of life and overall survival (OS). Myeloma bone disease (MBD) is a consequence of dysregulation of the fine balance of bone remodeling processes. Stimulated osteoclastic activity leading to bone resorption and suppressed osteoblastic activity, resulting in decreased bone formation, are key factors in the development of MBD [3]. Within the bone marrow microenvironment, interactions of MM plasma cells (MPCs) with bone marrow stromal cells (BMSCs) result in abundant secretion of various paracrine factors, such as TNF-α, IL-1β, IL-3, IL-6, IL-7, IGF-1, and VEGF, which in turn maintain survival and proliferation of MPCs. This vicious cycle plays a crucial role in the maintenance and development of MBD [4]. Beside the well-known RANK/RANKL/OPG system promoting increased osteoclastogenesis, there are factors involved in the downregulation of osteoblastic activity, such as inhibitors of the canonical Wnt/β-catenin pathway [5]. The canonical Wnt/β-catenin pathway is highly conserved and stimulated by different extracellular Wnt ligands, acting as autocrine/paracrine signals. Wnt ligands bind to specific Wnt membrane receptors and coreceptors. Via Wnt pathway stimulation, β-catenin is stabilized and transferred to the nucleus, resulting in the expression of genes involved in cell proliferation, survival, differentiation, and migration [6,7].

The major inhibitor of the Wnt/β-catenin signaling pathway is Dickkopf-related protein 1 (DKK-1), secreted by osteoblasts, BMSCs, and MPCs. Competitively binding to Wnt receptors, DKK-1 suppresses the differentiation of BMSCs into mature osteoblasts and blocks the maturation of osteoblasts, resulting in inhibition of osteoblastogenesis and formation of the bone mineral matrix [8,9]. Using oligonucleotide microarray technology, Tian at al. (2003) examined approximately 10,000 genes in plasma cells from MM patients with and without detectable osteolytic bone lesions. They found significant overexpression of four genes in plasma cells from patients with bone lesions, and only one of them (DKK-1 gene) codes for a secreted protein, implicated in the regulation of the Wnt signaling pathway. Increased expression of the DKK-1 protein was found in MPCs from patients with bone lesions, but not in those without bone lesions. There is evidence for strong correlation between DKK-1 transcripts in MPCs and DKK-1 levels in bone marrow plasma, and between DKK-1 in bone marrow plasma and DKK-1 in peripheral blood from MM patients. Moreover, in an experimental model with an uncommitted cell line of C2C12 mesenchymal stem cells was found an inhibition of osteoblast (oBs) differentiation when recombinant human DKK-1 was added [10]. The use of the anti-DKK-1 antibody (BHQ880) prevents MM-induced suppression of osteoblast numbers and increases the mineralizing surface and bone formation in 5T2MM murine model of MM [11]. In a recent study, it has been established that a novel antibody against the membrane Wnt-receptor stimulates Wnt-signaling and prevents the development of MM-induced bone resorption. By combining it with an anti-DKK-1 antibody, the authors have shown a more robust improvement in mice bone structure, thus providing a new strategy for bone disease treatment [12]. DKK-1 not only exerts inhibitory effects on osteoblastogenesis, but also may modulate RANKL and OPG production in osteoblasts, resulting in stimulation of osteoclastogenesis by increasing the RANKL/OPG ratio [13].

Another antagonist of Wnt/β-catenin signaling is the small monomeric glycoprotein sclerostin, encoded by the Sost gene, secreted from osteocytes and articular chondrocytes [14]. In a key paper of McDonald et al., it was shown that in MM, DKK-1 is expressed by MPCs, in contrast to sclerostin, which main source are osteocytes, but not MPCs [15]. As a member of the family of antagonists of bone morphogenic protein (BMP), sclerostin participates in the inhibition of bone formation. It was initially suggested that sclerostin acts by suppressing the BMP signaling pathway. Nowadays, it is proven that its main molecular mechanism is through binding the Wnt co-receptors LRP5/6, ultimately leading to suppression of the Wnt-pathway [16]. As a result, the differentiation of BMSCs is directed to an adipocyte phenotype, mature oBs are inhibited with impaired production of osteoblast-specific proteins, and their apoptosis is induced [17]. The negative effect on bone tissue is also enhanced by the stimulatory effect of sclerostin on RANKL production by osteocytes [18]. Data in literature on the role of sclerostin in MM are too scarce. It has been found that sclerostin is significantly higher in patients with fractures at diagnosis, as well as in patients with relapse, and correlates negatively with the marker of bone formation, specific bone alkaline phosphatase and positively with the C-telopeptide of collagen type-1, a marker of bone resorption [17]. Furthermore, animal model data have shown that administration of the anti-Scl antibody stimulates new bone formation and decreases osteolysis, thus providing new approaches to effectively control the MBD in patients with active MM [19]. In addition, the results from several clinical trials using neutralizing anti-sclerostin antibodies support the idea of recommending them as a valid therapeutic option in the treatment of osteoporosis [20].

It could be hypothesized that DKK-1 and sclerostin as inhibitors of bone formation could reflect the MBD severity and the effect of therapy. Therefore, we aimed to investigate the serum levels of DKK-1 and sclerostin in patients with MM at diagnosis and in the course of therapy, including autologous stem cell transplantation (ASCT).

## 2. Materials and Methods

### 2.1. Patients and Study Design

This prospective non-interventional study was conducted between June 2021 and December 2022. A total of 41 newly diagnosed MM patients (NDMM) and 33 age and gender-matched healthy controls were included in the study. The controls were healthy subjects who did not receive any steroid treatment. All patients were diagnosed according to the revised criteria of the International Myeloma Working Group [21]. The stage was determined according to the International Staging System, ISS [22]. Evaluation of bone disease was performed by whole-body low-dose computed tomography (WBLDCT) imaging, and according to the bone damage, the patients were divided into two groups: G1 (≤3 lesions) and G2 (>3 lesions and/or presence of bone fractures). All patients received chemotherapy according to the VCD protocol (Velcade—1.3 mg/m^2^ on days 1, 4, 8, and 11; Cyclophosphamide—400 mg/m^2^ on days 8 and 15, Dexamethasone—40 mg on days 1–4 and 8–11 per cycle, repeated every 28 days) and Zoledronic acid. Only three patients with creatinine levels >265 μmol/L did not receive Zoledronic acid. The response to treatment was assessed according to the current International Unified Response Criteria in MM [23]. After four cycles of treatment, patients up to 65 years of age, achieving very good partial response (VGPR) and complete response (CR), proceeded towards ASCT, and patients above 65 years of age in stable disease (SD) or achieving partial response (PR) continued with another four cycles of VCD.

Levels of bone biomarkers DKK-1 and sclerostin were assessed at diagnosis (T0) and at different time points in the course of treatment: after four cycles of chemotherapy (timepoint T1), after another four cycles (eight cycles) of chemotherapy (timepoint T2), and three months after ASCT (timepoint TA). The window period for each time point was ±3 days.

The study was approved by the local Ethical Committee (Protocol No 94/25 June 2020). Written informed consent was obtained from all participants before starting the study.

### 2.2. Methods

After venipuncture, fasting venous blood was drawn in serum and EDTA vacutainers. The routine biochemical parameters, such as total protein (TP), albumin, ß2-microglobulin (Β2M), creatinine, and calcium, were measured with an automatic analyzer (ADVIA 1800 Chemistry System, Erlangen, Germany), and a complete blood count was performed using an automated haematology analyzer, Sysmex XN-9000. For determination of the proteins Dickkopf-1 (DKK-1) and sclerostin, blood serum was separated within 1h and aliquots were stored at −80 °C until measurement by commercial ELISA kits based on competitive monoclonal antibody assay (Sunredbio, Shanghai, China), strictly following the manufacturer’s instructions. According to the producer, the sensitivity of the DKK-1 kit was 0.979 ng/mL with the standard range set from 12.51 ng/mL to 400 ng/mL. The intra-assay CV was <9% and the inter-assay CV was <11%. The sensitivity of the sclerostin kit was 37.5 ng/mL with an assay range from 62.5 ng/mL to 4000 ng/mL. Bone marrow aspiration was done for morphological evaluation of bone marrow infiltration (BMI) by MM plasma cells (MPCs).

### 2.3. Statistical Analysis

Results were presented as mean ± standard deviation (SD), median and interquartile range (IQR: 25–75th percentile), or number (*n*) and percentage (%), as appropriate. Data analysis was performed using GraphPad Prism v. 8.0.1, San Diego, CA, USA. Standard statistical methods were used for descriptive statistics, unpaired Student’s *t*-test for normally distributed parameters and the Mann–Whitney U-test for non-normally distributed interval data for the comparison of groups. Spearman’s correlation analysis was performed to evaluate the relationships between continuous variables. For all tests, *p*-values < 0.05 were considered as statistically significant.

## 3. Results

The baseline characteristics of patients enrolled in the study are shown in Table 1. Regarding ISS staging, approximately 40% of patients were in stage I (*n* = 16), around 15% in stage II (*n* = 6), and 46% in stage III (*n* = 19). According to their bone disease status, nine patients were included in the G1 group, and 32 patients were in the G2 group. More than 50% of patients were with IgG type M-protein, only 15% of them were with IgA type M-protein, and about 30% were free light chain (FLC) type monoclonal protein. Assessing the bone marrow aspirates, MPCs infiltration below 60% was found in 41.5% of NDMM patients and above 60% in 58.5% of MM cases.

The results of some routine laboratory parameters of MM patients at baseline are presented in Table 2.

### 3.1. Sclerostin and Dickkopf-1 Assessed in NDMM Patients at Baseline (T0)

**Figure 1:**
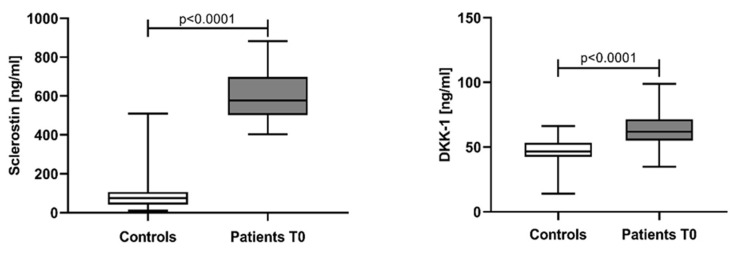
Serum levels of sclerostin and DKK-1 in controls and patients at baseline. Statistical significance was indicated at *p* < 0.05.

In NDMM patients, the median serum levels and IQR for sclerostin and DKK-1 at baseline were 576.8 ng/mL (502.1–697.8) and 61.75 ng/mL (55.11–71.45), respectively, significantly higher than those in controls: 74.76 ng/mL (41.83–106.2) for sclerostin and 46.62 (42.43–53.32) for DKK-1 (Figure 1).

**Figure 2:**
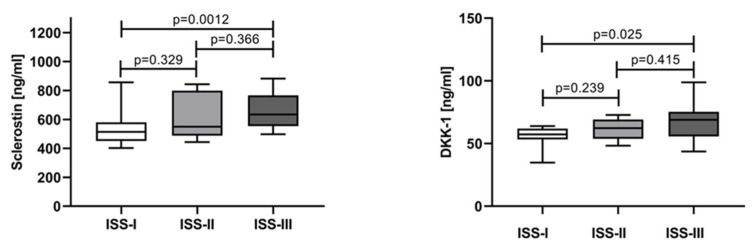
Comparison of serum levels of sclerostin and DKK-1 in NDMM patients, stratified according to the ISS staging system. Statistical significance was indicated at *p* < 0.05.

The serum levels of sclerostin and DKK-1 gradually increased with the disease stage. The median and IQR for sclerostin in patients in ISS-I, ISS-II, and ISS-III were 513.7 ng/mL (451.5–579.6), 550.1 ng/mL (488.0–798.4), and 634.7 ng/mL (554.4–756.9), respectively. The same phenomenon was observed for DKK-1: the medians for ISS-I, ISS-II, and ISS-III were 57.7 ng/mL (53.29–61.9), 62.32 ng/mL (53.82–69.24), and 68.94 ng/mL (55.72–75.34), respectively. For both proteins, statistical difference was reached between ISS-I and ISS-III (Figure 2).

**Figure 3:**
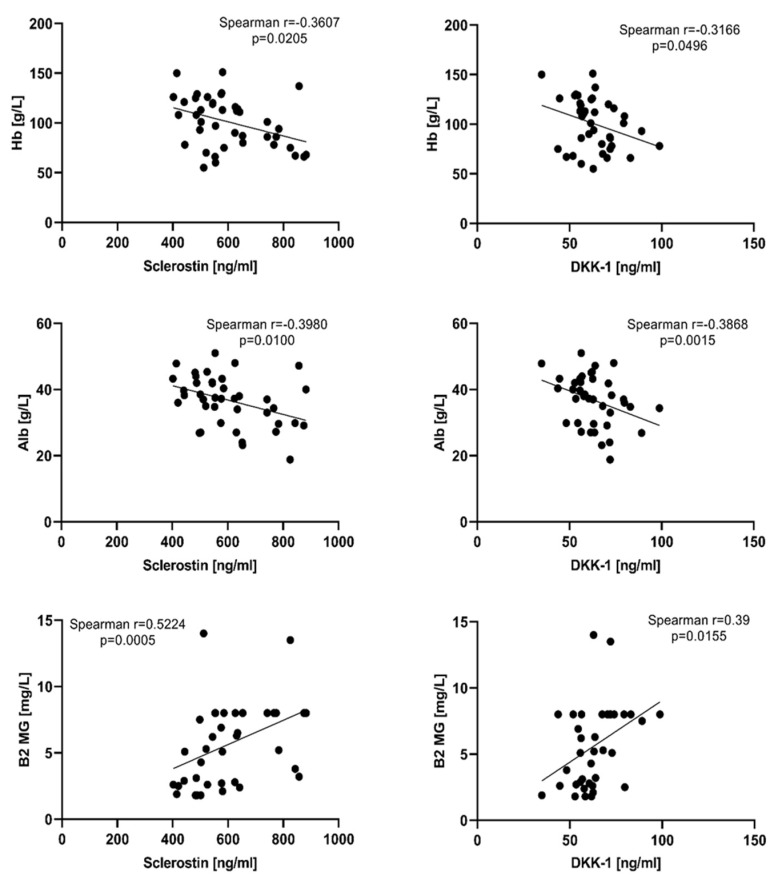
Correlations of DKK-1 and sclerostin with Hb, albumin, and B2MG. Hb–hemoglobin; Alb—albumin, Β2MG—ß2 microglobulin. Statistical significance was indicated at *p* < 0.05.

To evaluate the relationship between the tested bone markers and some routine laboratory parameters, Spearman correlation analysis was performed. Weak negative correlation was found with hemoglobin and albumin for both sclerostin and DKK-1, while with ß2 microglobulin the association was moderate and positive (Figure 3). Statistically significant positive correlation was found for sclerostin and total protein, creatinine, and BMI (r = 0.3323, *p* = 0.0338; r = 0.4799, *p* = 0.0015; r = 0.4527, *p* = 0.0030, respectively). Such correlations with the aforementioned laboratory parameters were not found for DKK-1.

**Figure 4:**
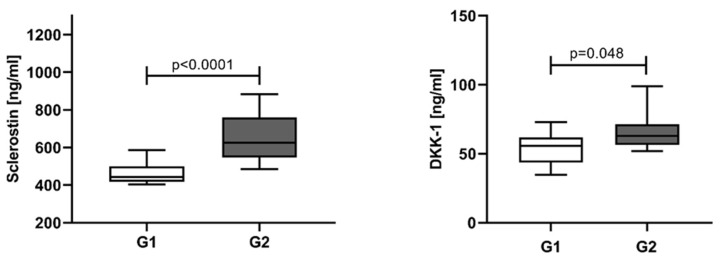
Serum levels of sclerostin and DKK-1 in NDMM patients stratified by the number of bone lesions. G1—patients with ≤3 osteolytic lesions, G2—patients >3 osteolytic lesions and/or presence of bone fractures. Statistical significance was indicated at *p* < 0.05.

The NDMM patients were divided into two groups according to the number of bone lesions: G1 ≤ 3, and G2 with more than three osteolytic lesions and/or bone fractures. An elevation in median sclerostin levels was found in the G2 group (625.50 ng/mL, IQR 547.0–760.0) compared to G1 (443.6 ng/mL, IQR 417.7–499.8). Similar results were obtained for DKK-1, higher levels in G2 (62.86 ng/mL, IQR 56.36–71.45) and lower in G1 (55.68 ng/mL, IQR 43.71–61.75) (Figure 4).

**Figure 5:**
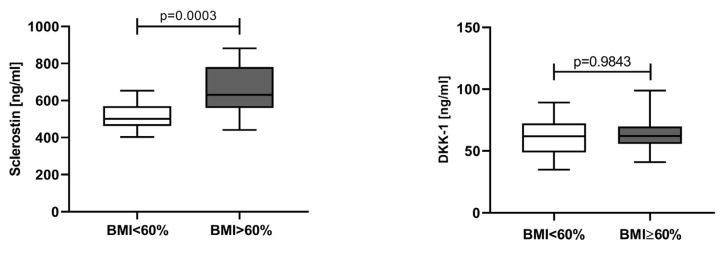
Serum levels of sclerostin and DKK-1 in NDMM patients, stratified according to BMI by MPCs. BMI—bone marrow infiltration; MPCs—multiple myeloma plasma cells. Statistical significance was indicated at *p* < 0.05.

To evaluate the impact of tumor burden on the serum sclerostin and DKK-1, the patients were divided into two groups using a cut-off of 60% MPCs [24]. Bone marrow MPCs below 60% were found in 41.5% of NDMM patients and their serum levels of sclerostin were significantly lower (501.4 ng/mL, IQR 462.9–569.8 ng/mL) than those in patients with MPCs ≥ 60% (median 630.5 ng/mL, IQR 559.6–781.2 ng/mL). Surprisingly, such difference was not indicated for DKK-1 (MPCs < 60%: 61.75 ng/mL, IQR 48.76–72.30 ng/mL; MPCs ≥ 60%: 62.08 ng/mL, IQR 55.67–69.78 ng/mL) (Figure 5).

### 3.2. Sclerostin and Dickkopf-1 Assessed at Different Time Points and According to the Response of Treatment

**Figure 6:**
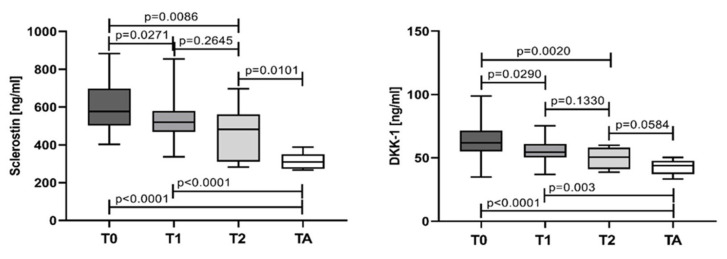
Sclerostin and Dickkopf-1 assessed at different time points. T0—at diagnosis; T1—after 4 cycles of chemotherapy; T2—after another 4 cycles (8 cycles) of chemotherapy; TA—after autologous stem cell transplantation. Statistical significance was indicated at *p* < 0.05.

Serum values for both sclerostin and DKK-1 gradually decrease in the course of treatment. For both parameters, significant difference was established between values measured at T0 (sclerostin: 576.8 ng/mL, IQR 502.1–697.8; DKK-1: 61.75 ng/mL, IQR 55.11–71.45) versus those at T1 (sclerostin: 519.5 ng/mL, IQR 469.1–580.6; DKK-1: 54.57 ng/mL, IQR 50.32–61.04), T2 (sclerostin: 481.6 ng/mL, IQR 311.3–561.5; DKK-1: 50.55 ng/mL, IQR 41.09–58.13), and TA (sclerostin: 309.8 ng/mL, IQR 273.0–350.1; DKK-1: 43.9 ng/mL, IQR 37.2–47.74). The degree of significance increases in the course of treatment, more pronounced in T2 and TA (Figure 6).

Table 3 summarizes the frequency distribution of patients according to their treatment response at all treatment points. Complete and very good partial response was achieved in 45.5% of patients at T1, followed by 27.3% at T2. The highest percentage of complete response was achieved after ASCT.

**Figure 7:**
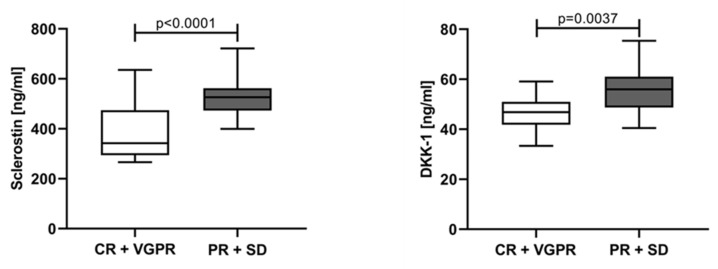
Sclerostin and Dickkopf-1 according to the treatment response. CR—complete response, VGPR—very good partial response, PR—partial response, SD—stable disease, PD—progressive disease. Statistical significance was indicated at *p* < 0.05.

To evaluate the variations in sclerostin and DKK-1 according to treatment response, the patients were categorized into two groups: (1) patients in CR and VGPR regardless of the treatment point (T1 + T2 + TA) at which they achieved it; and (2) patients with PR and SD. Both tested parameters revealed significantly lower values in the group with CR and VGPR (sclerostin 372.2 ng/mL, IQR 310.1–503.2; DKK-1 50.39 ng/mL, IQR 41.48–57.57) compared to the patients with PR, SD, and PD (sclerostin 536.4 ng/mL, IQR 469.1–582.7; DKK-1 57.72 ng/mL, IQR 50.37–61.04) (Figure 7). In addition, patients with complete or very good response revealed significantly lower levels for both tested parameters vs. baseline levels (*p* < 0.0001).

## 4. Discussion

Interaction of MPCs with the bone marrow microenvironment plays an important role in the disruption of otherwise well-balanced processes of bone remodeling. The increase in the tumor burden is a prerequisite for the formation of osteolytic lesions [4]. Sclerostin and DKK-1, as inhibitors of osteoblastogenesis, are one of the pathogenetic factors underlying the MBD [3]. In the present study, the serum levels of DKK-1 and sclerostin in symptomatic NDMM patients with MBD were significantly higher compared to the controls. In regard to DKK-1, our results are consistent with the data of other studies [25,26,27]. The overexpression of DKK-1 by MPCs was proven initially by Tian et al. [10]. They demonstrated a positive correlation between DKK-1 in bone marrow plasma and peripheral blood from MM patients. Thereafter, Politou et al. revealed that DKK-1 serum levels in 32 NDMM patients were significantly higher not only compared to the controls, but also to patients with monoclonal gammopathy of undetermined significance (MGUS). Based on these findings, the authors hypothesize that although osteoclast activity is increased in patients with MGUS, it is compensated by still-normal osteoblast function, and lytic lesions do not develop [25]. Some researchers underscored the importance of DKK-1 as an inducer for the Sost gene, thus partly explaining the mechanism of sclerostin biosynthesis in immature osteoblasts while inhibiting osteoblast differentiation [28,29]. Terpos et al. established that circulating sclerostin of symptomatic NDMM was significantly increased in comparison to controls [27], later confirmed in clinical settings for MM patients compared to both healthy controls and MGUS patients, but also in murine models [29]. In contrast, Brunetti et al. did not prove the difference in sclerostin serum levels between MGUS patients versus symptomatic NDMM patients. On the other hand, they demonstrated the increased expression of sclerostin mRNA in freshly purified CD138+ cells from bone marrow aspirates of MM patients compared to undetectable expression in CD138+ cells from MGUS controls [30].

We revealed the increase of DKK-1 and sclerostin serum levels with the advancement of the ISS stage. The concentrations for both parameters in ISS-II were higher than those in ISS-I and lower than those in ISS-III without yet reaching statistical significance. A significant difference in DKK-1 and sclerostin levels was found only between ISS-I and ISS-III. We also observed positive correlation between DKK-1 and sclerostin serum levels with ß2-microglobulin and negative correlation with albumin as the main indicators for ISS stratification in MM. Our results are in accordance with the findings of Politou et al., demonstrating significant differences in DKK-1 between NDMM patients in ISS-I versus those in ISS-II and ISS-III, as well as in ISS-II + ISS-III [25]. Similar results were obtained by Terpos et al., not only for DKK-1, but also for sclerostin serum levels in MM patients [17,27]. In addition, Feng et al. found that patients with low DKK-1 revealed significantly longer survival times than those with high DKK-1 [31]. With regard to sclerostin, the negative correlation between its serum levels and OS has already been established by Terpos et al. [17]. Similarly, Wang et al. observed significantly shorter median survival in MM patients with high sclerostin levels in bone marrow plasma than those with low sclerostin levels [32].

One of the main characteristics of the Wnt inhibitors is that they contribute to the development of lytic bone lesions by impairing OBs differentiation. We have demonstrated significantly higher DKK-1 and sclerostin levels in patients with >3 osteolytic lesions and/or presence of bone fractures in comparison to those in patients with ≤3 osteolytic bone lesions. These findings confirm the notion that DKK-1 and sclerostin levels reflect well the bone disease status. Concerning this aspect, there are conflicting results obtained in different studies. The first ones who revealed correlation between serum concentration of DKK-1 and MBD were Kaiser et al. They found that patients with at least one lytic bone lesion had five-fold elevated serum DKK-1 concentrations when compared to those without lytic lesions [26]. Terpos et al. confirmed these findings—patients with lytic disease had a statistically significant increase of DKK-1 concentrations compared to patients with no lytic disease [27]. On the contrary, Politou at al. did not find a correlation between serum DKK-1 concentrations and the extent of bone disease. According to the authors, this result can be explained by the relatively small number of patients and/or by the limitations of the imaging technique (plain radiography) used to detect the osteolytic lesions [25]. Consistent with our results regarding sclerostin levels in MM patients, Brunetti et al. found almost two-fold higher, although nonsignificant, values in patients with MBD vs. those without bone lesions [30]. Similarly, Terpos et al. showed a borderline increase in serum sclerostin in patients with advanced lytic disease [27]. In support to the notion that sclerostin levels reflect MBD are the findings of Wang et al. for significantly higher sclerostin concentrations in bone marrow plasma in patients with more than three osteolytic bone lesions compared to patients with 0–3 lesions [32].

Until recently, the prognostic impact of MPCs percentage was not well established. In 2020, Al Saleh et al. proved that MPCs ≥ 60% at diagnosis of MM was predictive for PFS and OS both in low- and high-risk patients [24]. To the best of our knowledge, there is no data in the literature concerning the relationship between serum concentrations of Wnt-pathway inhibitors and MPCs infiltration in the bone marrow. It was curious to assess the impact of MPCs bone marrow infiltration on sclerostin and DKK-1 serum levels in NDMM patients. The sclerostin values were significantly lower in patients with BMI <60% than those in patients with BMI ≥ 60%. Moreover, we found a moderate positive correlation between sclerostin and BMI by MPCs. Thus, our results support the idea that serum sclerostin levels could be used as serum predictor of disease progression. Moreover, in a recent study of Mabille et al. [33], an increase of DKK-1 and sclerostin levels was established several months before the MM relapse. The authors consider this increase of DKK-1 and sclerostin as an early phenomenon during relapse, preceding MPCs proliferation or excretion of the monoclonal component. Interestingly, for DKK-1 serum levels we did not observe a difference between the above-mentioned groups, and there was no correlation between DKK-1 and BMI. The crosstalk between bone marrow environment with MPCs is mediated by several cell–cell interactions and paracrine/autocrine mediators. In their research, Eda et al. had showed that MPCs have the ability to stimulate sclerostin expression in OBs via secretion of DKK-1 [29]. It could be speculated that minimal local increase of DKK-1 in bone marrow could enhance the sclerostin biosynthesis in an exponential way, leading to a more pronounced elevation of sclerostin in blood/serum.

One of the components of the induction protocol in our study was bortezomib with proven anti-tumor efficacy. As a proteasome inhibitor, it not only effectively kills MPCs, but also improves bone remodeling by inducing osteoblast differentiation, and thus inhibiting the osteolytic progression in MM patients [34]. According to our protocol, a bortezomib-based regimen (VCD) was used for induction. During the course of treatment, a significant decrease in levels of sclerostin and DKK-1 were measured at all time points (T1, T2, and TA) in comparison to baseline. Patients non-eligible for ASCT showed a 7.3% decrease in serum sclerostin and a 7.4% decrease in DKK-1 in T2 vs. T1, although statistical significance was not reached. Several studies have explored the influence of chemotherapy on DKK-1 and sclerostin, with conflicting data. Heider at al. demonstrated for the first time that serum DKK-1 decreases in MM responders, regardless of the treatment regimen. The authors have suggested that MPCs are the main source of circulating DKK-1 [35]. This finding was confirmed by Terpos et al. only for DKK-1 and not for sclerostin. Serum DKK-1 levels decreased significantly after treatment compared to baseline and reached the control levels. In contrast, circulating sclerostin levels in MM patients at the plateau phase increased in comparison to controls as well as to baseline [27,36]. On the other hand, four cycles of Bortezomib monotherapy reduced significantly the sclerostin levels in both responders and non-responders in relapse settings, revealing another mechanism of action of bortezomib in reversing osteoblast dysfunction in myeloma. According to the authors, the anti-myeloma effect of bortezomib is due to its reducing effect on DKK-1, which enhances sclerostin expression, or may be due to an independent effect of bortezomib on osteocytes [17].

ASCT-eligible patients were tested for sclerostin and DKK-1 three months after transplantation, and their levels were found to be significantly lower than those measured in all timepoints (T0, T1, and T2). The main advantage of high-dose chemotherapy followed by ASCT is the significant reduction of tumor burden in a high percentage of patients with deeper response achieved. As a result, the sclerostin and DKK-1 levels after ASCT were significantly lower than those observed in T2. Moreover, DKK-1 levels after ASCT even reached the control levels. Most of the studies showed similar results regarding DKK-1. Politou et al. proved that statistically reduced values of DKK-1 after transplantation were sustained for several months, and a restoration of osteoblastic function was observed [25]. Lemaire et al. observed also a significant decrease in DKK-1 six months after ASCT [37]. In a more recent study on NDMM patients undergoing ASCT, followed by bortezomib and lenalidomide consolidation without bisphosphonates, was found a significant decrease for sclerostin, but not for DKK-1. The authors speculated that the lack of changes in DKK-1 during the consolidation phase of treatment is probably due to its huge reduction during the bortezomib-based induction phase of treatment with coadministration of bisphosphonates [38].

Additionally, we have demonstrated that the decrease of serum levels of sclerostin and DKK-1 correlated with the depth of the response. Patients with CR and VGPR had significantly lower levels compared to PR and SD. Moreover, bad responders (PR + SD) had values for both proteins that did not differ from those measured at baseline. These findings support the idea that a successful treatment, decreasing the tumor burden, leads to a significant decrease of Wnt inhibitors, perhaps an essential prerequisite for restoration of the fine balance between bone remodeling processes. Studying the effect of different treatment regimens for MM on serum DKK-1, Heider at al. revealed that a significant decrease of DKK-1 serum concentration was seen only in responders (patients achieving CR or PR), but not in non-responders, irrespective of the regimen [35]. The three-year follow-up study on 39 MM patients after ASCT showed that good responders (defined by at least 75% decrease of serum paraprotein or light chain level) had significant decrease in DKK-1 levels six months after ASCT, not observed in bad responders. An interesting finding was that 12 months after transplantation, patients with late relapse had a non-significant decrease in DKK-1 levels versus pre-ASCT levels. Moreover, patients with rapid relapse, defined by the necessity to start treatment again less than 18 months after ASCT, had a substantial increase in DKK-1 levels [37]. The data for sclerostin are relatively scarce.

This study evaluates the role of sclerostin and DKK-1 as good indicators for the severity of MBD at diagnosis and for monitoring the MM patients in the course of treatment. As responsible factors for the suppressed osteoblasts activity, DKK-1 and sclerostin become a promising molecular target for the treatment of MBD [39]. Romosozumab, an anti-sclerostin antibody, has been approved for treatment of osteoporosis and might be an effective drug in limiting MBD. Moreover, a phase 1 clinical trial evaluating the efficacy and safety of Romosozumab (NCT05775094) started this year in MM settings.

The current study has several limitations: the low number of patients, which is a limitation for additional subgroup analysis, and the short follow-up for survival assessment. As serum often does not reflect well local changes in the bone, a potential limitation of the study is the lack of bone marrow plasma assessment for sclerostin and DKK-1 levels.

## 5. Conclusions

Destructive bone lesions are the hallmark of MM due to the imbalance in the bone-remodeling processes with a prevalence of osteoclastic over osteoblastic activity. In the last decade, high attention has been paid not only to increased osteoclastogenesis, but also to decreased osteoblastogenesis in MBD. Growing literature data on both sclerostin and DKK-1 reveals that they are key modulators of impaired osteoblast function in MM. In the present study, the serum levels of sclerostin and DKK-1 in NDMM patients reflect the severity of MBD, the ISS stage, and the response to treatment. ASCT leads to the most prominent decrease in the serum levels of both proteins, reaching the values of controls. Exploring serum levels of sclerostin and DKK-1 could represent a new tool for better monitoring of the disease.

## Figures and Tables

**Table 1:** Patients’ and controls’ characteristics at baseline.

Characteristic	*n* (%)
Patients	41 (100%)
Controls	33 (100%)
**Age (years)**	
Patients	64.02 ± 12.14 *
Controls	60.64 ± 7.56 *
Gender **:	
Patients (male/female)	20/21 (48.78%/51.22%)
Controls (male/female)	18/15 (54.54%/45.46%)
**ISS stages:**	
I	16 (39.02%)
II	6 (14.63%)
III	19 (46.34%)
**Bone disease status:**	
G1 (≤3 osteolytic lesions)	9 (21.95%)
G2 (>3 osteolytic lesions + bone fractures)	32 (78.05%)
**M-protein type:**	
IgG	23 (56.10%)
IgA	6 (14.63%)
FLC (κ + λ)	12 (29.27%)
**MPCs infiltration in bone marrow:**	
<60%	17 (41.46%)
>60%	24 (58.54%)

ISS—International Staging System; Ig—immunoglobulin; FLC—free light chain; MPCs—myeloma plasma cells; (*—*t*-test was done (*p* = 0.1662) to compare patients’ and controls’ ages, presented as mean ± SD); **—Chi-square analysis was done (χ^2^ = 0.2432, *p* = 0.6219) to compare gender distribution in patients and the control group; statistical significance was indicated at *p* < 0.05.

**Table 2:** Patients’ results from routine laboratory testing at baseline.

Parameters	Mean ± SD, (Range)
Hb (g/L)	100.78 ± 25.36 (55–151)
WBC (×10^9^/L)	6.40 ± 2.95 (2.03–14.58)
Plt (×10^9^/L)	208.41 ± 103.31 (32–461)
Creatinine (µmol/L)	126.05 ± 91.53 (53–449)
LDH (IU/L)	425.76 ± 370.34 (200–2571)
Total protein (g/L)	92.70 ± 20.99 (55–133)
Albumin (g/L)	36.71 ± 7.58 (19–51)

Hb—hemoglobin; WBC—white blood cells, Plt—platelets; LDH—lactate dehydrogenase; Β2MG—ß2 microglobulin.

**Table 3:** Treatment response of patients at different treatment points.

Treatment Response	T1 (*n* = 22)	T2 (*n* = 11)	TA (*n* = 10)
CR, *n* (%)	6 (27.3%)	-	9 (90%)
VGPR, *n* (%)	4 (18.2%)	3 (27.3%)	1 (10%)
PR, *n* (%)	8 (36.4%)	5 (45.5%)	-
SD, *n* (%)	3 (13.6%)	3 (27.2%)	-
PD, *n* (%)	1 (4.5%)	-	-

CR—complete response; VGPR—very good partial response; PR—partial response; SD—stable disease; PD—progressive disease; T0—at diagnosis; T1—after 4 cycles of chemotherapy; T2—after another 4 cycles (8 cycles) of chemotherapy; TA—after autologous stem cell transplantation. Statistical significance was indicated at *p* < 0.05.

## Data Availability

Not applicable.
